# A comprehensive review of machine learning techniques on diabetes detection

**DOI:** 10.1186/s42492-021-00097-7

**Published:** 2021-12-03

**Authors:** Toshita Sharma, Manan Shah

**Affiliations:** 1 Department of Electronics and Communication Technology, Nirma University, 382481 Ahmedabad, Gujarat India; 2 Department of Chemical Engineering, School of Technology, Pandit Deendayal Energy University, 382426 Gandhinagar, Gujarat India

**Keywords:** Machine learning, Deep learning, Health care, Diabetes detection

## Abstract

Diabetes mellitus has been an increasing concern owing to its high morbidity, and the average age of individual affected by of individual affected by this disease has now decreased to mid-twenties. Given the high prevalence, it is necessary to address with this problem effectively. Many researchers and doctors have now developed detection techniques based on artificial intelligence to better approach problems that are missed due to human errors. Data mining techniques with algorithms such as - density-based spatial clustering of applications with noise and ordering points to identify the cluster structure, the use of machine vision systems to learn data on facial images, gain better features for model training, and diagnosis via presentation of iridocyclitis for detection of the disease through iris patterns have been deployed by various practitioners. Machine learning classifiers such as support vector machines, logistic regression, and decision trees, have been comparative discussed various authors. Deep learning models such as artificial neural networks and recurrent neural networks have been considered, with primary focus on long short-term memory and convolutional neural network architectures in comparison with other machine learning models. Various parameters such as the root-mean-square error, mean absolute errors, area under curves, and graphs with varying criteria are commonly used. In this study, challenges pertaining to data inadequacy and model deployment are discussed. The future scope of such methods has also been discussed, and new methods are expected to enhance the performance of existing models, allowing them to attain greater insight into the conditions on which the prevalence of the disease depends.

## Introduction

Given the growing population, it is necessary to develop systems to augment health and mitigate increasing concerns around the world. As scientific research continues to advance, the development of such system is becoming more efficient. Healthcare systems are designed to provide people with the requirements for good health and perform the detection and diagnosis of disease and conditions correctly with greater efficiency, as proposed in the conventional methods. In general patients are often highly concerned as to the quality of healthcare system and facilities available to provide treatment. The benefits of improvements in healthcare systems tend to affect people who have prevailing ailments more directly, and this group comprises the majority of the group of people affected by many diseases such as diabetes, blood sugar, and blood pressure issues [1]. According to the National Diabetes Statistics Report 2020, every 1 in 10 people in United States have diabetes, and new cases of diabetes 1 and 2 have significantly increased among young people. As health and healthcare form a critical pillars of a healthy society, it is necessary to use the capabilities of computational methods and artificial intelligence [2] to develop new methods for application in healthcare systems to promote a healthier society and reduce the risk of such diseases in our generations, further increasing the quality of life.

There has been a huge impact in the medical world with the advancement of technology. Health outcomes may depend on a matter of seconds for individuals who may not be able to reach a hospital or receive emergency treatment. Technology bridges this gap in distance and resources for all people to whom its benefits are extended. Various technologies have been developed using magnetic resonance imaging machines in video technology. Internet-based applications can provide patients with customizable services. After a few clinical visits, the remainder of the work can be fulfilled through high-tech services such as telehealth. Clinicians can communicate with patients through the Internet to better serve their needs [3]. One example of the use of video technology involves the provision of such mechanization in case of emergency to patients in trauma in rural and urban areas where clinical care may be unavailable [4]. Technology has the capacity to enable home healthcare with better productivity and security [5]. Data accuracy and availability have been proposed as among the most significant problems faced by hospitals, which involve maintaining and further processing patient data. Various algorithms in the field of machine learning and deep learning have been beneficially applied in practice to medical treatments. Some state-of-the-art ideas emerge from the massive implementation of technologies such as the creation of matching algorithms and natural language processing [6]. Data mining can be used to extract data directly, instead of relying on expert knowledge. These methods are considered to produce unique and distinctive patterns to create personalized plans for each hospital [7].

Diabetes mellitus (DM) is one of the most archetypal diseases worldwide. It is a disease that implies that a person’s body systems are unable to work efficiently to use energy from food. There are four major types of diabetes known, including type-1, type-2, gestational, and other forms, with the most common being type-1 and type-2 [8]. Type-1 diabetes usually occurs in the young age group of 30–40 and is insulin dependent. Patients are required to take in doses of insulin for entire lives. In contrast type-2, predominates among the over-40 age cohort and is often related patients’ weight. Type-2 diabetes is known to have greater prevalence globally, accounting for more than 90 % of cases [9]. Because this disease has long been near the top of global rankings listing serious of diseases, many researchers and doctors have proposed algorithms and methods for its treatment and detection. The implementation of these algorithms is rooted in the disciplines of deep learning and machine learning. With predictive analysis supported by neural networks, specifically convolutional neural networks (CNNs) and recurrent neural networks (RNNs), such methods have the ability to determine sentiments and learn high model features automatically [10, 11]. Some researchers have implemented the use of machine learning through algorithms such as gradient boosted trees, which can be used to create predictive models of the progression from prediabetes to diabetes, and have aimed to provide early diagnosis for better treatment and to reduce further risks [12]. Another study applied a modified support vector machine (SVM) algorithm as an efficient method for both linear and non-linear data [13].

Since the advent of artificial intelligence and related technologies, such computational methods have been applied to real-time detection models in almost every field. The use of data mining, machine learning, deep learning, and computer vision has drastically reduced the difficulty of studying newer techniques that can significantly improvement of existing methods. In the next section, the algorithms and methods are surveyed.

### Application of latest technology in diabetes detection

The algorithms used in data mining, machine learning, or any field of artificial intelligence perform predictive modeling, that is, the use of data and statistics to predict future outcomes based on historical data. The most common symptoms of diabetes include abnormal metabolism, hyperglycemia, and an associated risk for specific complications affecting the eyes, kidneys, and nervous system, which are major parts of the body. Such symptoms are used to gather data, and then the modeling is performed based on age and gender categories. One such algorithm is ordering points to identify cluster structure (OPTICS), which is set of ordering points to identify clustering structures. OPTICS is an advanced version of density based spatial clustering of applications (DBSCAN) with noise, and it eliminates all negative aspects of DBSCAN. The data clustering method used in this algorithm is a balanced iterative reducing and clustering algorithm using hierarchies (BIRCH) that selects the most suitable data for further analysis. Thus, the naïve Bayes (NB) data mining technique is used, and BIRCH and OPTICS are used for clustering similar types of data and used for identification of the correct algorithm for better accuracy [14]. Apache Spark is among the fastest growing platforms for health analysis. It operates more rapidly than the Hadoop platform, making it more easily usable and applicable to clinical practice [11].

Another such application in the field of OpenCV is the use of the computer-assisted non-invasive DM detection system. This system provided immediate results from facial images. The model examined four health blocks of the skin: forehead, left cheek, right cheek, and nose bridge. Then, feature extraction was performed using a local binary pattern and then classification using the *k*-nearest neighbor (KNN) clustering and a SVM. All these features were connected with software and show the results in real time [15].

Islam et al. [16] used different algorithms for a diabetes symptom dataset, include NB, random forest (RF), logistic regression (LR), and a decision tree (DT). First, the dataset with the patient system is entered into the system on which predictive algorithms are applied. After this, the dataset is input to the database and the performance or accuracy achieved by the model is observed. The most suitable algorithm is commonly selected based on the highest accuracy and best performance. The user’s data is again taken as the input for the algorithm for further training and evaluation to increase the accuracy of the model in real time.

Irido-diagnosis is a predictive system in which the disease is detected through iris patterns. This was used for the detection of diabetes via the following method. Data were gathered from diabetic and non-diabetic patients on which pre-processing was performed. The next part is called image segmentation, wherein the iris of the eye is separated from the image in the dataset. Normalization is performed wherein the circular iris is converted to a rectangle using polar mapping. Feature extraction is then performed on this final image. A gray-level co-occurrence matrix is used to characterize images, which is a process for examining the texture. This process assigns numbers from 1 to 8 to images areas by calculating how often pairs of pixels with specific values and in a specified spatial relationship occur in the image. In this matrix, features such as contrast, correlation, dissimilarity, homogeneity, variance, and entropy are the features are pertinent to provide high-quality data.

These are just some of the authors among many who have contributed to the literature in this field. Below, a detailed survey of the methodology used by other researchers in this field is provided.

### Machine learning in diabetes detection

Machine learning is a method by which a computational system learns the features of input data. Such methods haves proven effective for the detection of diabetes. Many machine learning algorithms have been developed, including supervised, unsupervised, and reinforcement learning methods. This is evidently practical because machine learning methods are driven by data. With such massive amounts of data fed into the database, machine learning can save considerable human effort. Models are trained on this data and provide the most suitable output based on the input data. The models can be trained on any parameters that are feasible for practicality and medical requirements. Some might examine facial features, while others look for blood report data obtained from patients. Because there are many symptoms of the disease, the parameters vary accordingly. With many proposed methods, researchers have probed various algorithms and tweaked numerous hyperparameters to obtain results that seem most suitable for real-life applications.

**Fig. 1:**
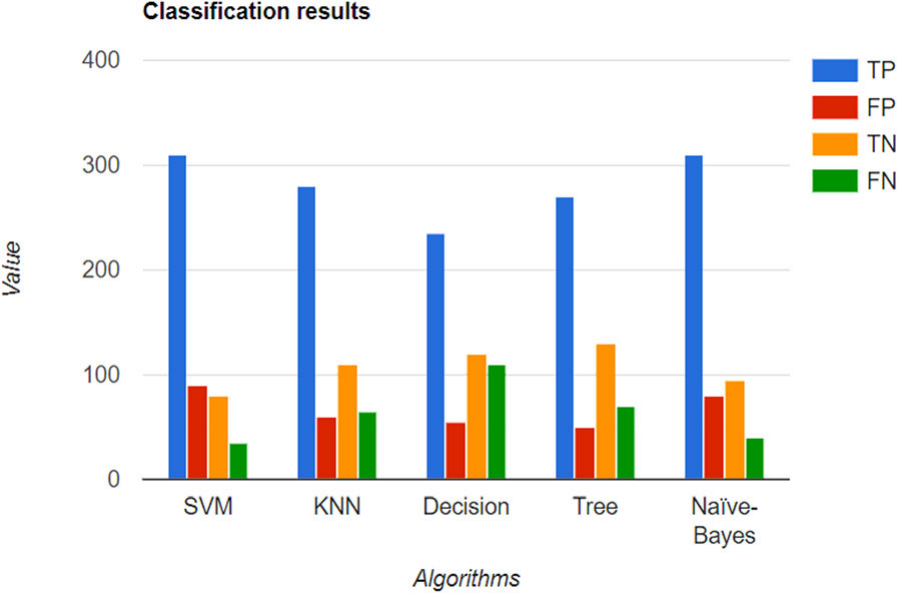
Classification results of SVM, KNN, NB, DTs and LR in form of true positive (TP), false positive (FP), true negative (TN) and false negative (FN) which are the parameters of confusion matrix [17]

Choudhury and Gupta [17] used different algorithms to classify people into two categories: high- and low-risk individuals. They used a SVM to establish a hyperplane for categorization, a KNN classification technique for clustering new data into groups, DTs, RF and NB classifiers, and the binary classifier method called LR. On comparing the accuracies for this classification in the form of a confusion matrix, as shown in Fig. 1, the LR algorithm was found to be the most efficient and accurate, while the DT algorithm, achieved the lowest accuracy.

**Fig. 2:**
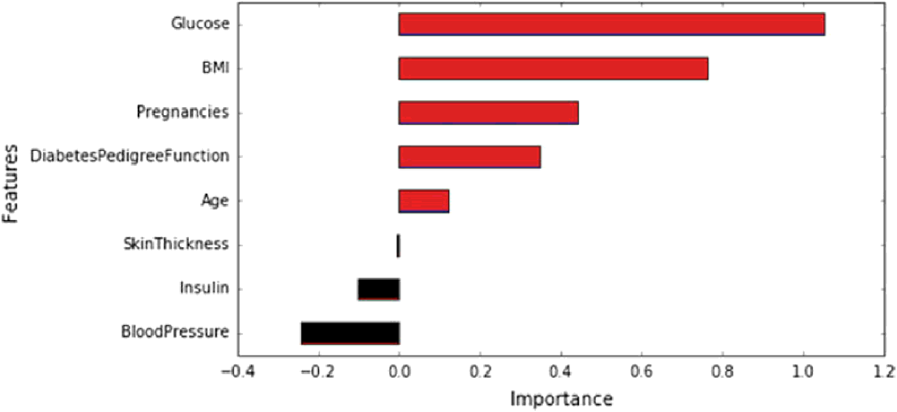
The weight of each of the features which yield the result variable [18]

Shukla [18] used a LR algorithm, took out a dataset that showed the maximum accuracy would be yielded if parameters such as glucose, body mass index (BMI), and pregnancies, were used, which were represented in the form of a bar chart, as shown in Fig. 2. The author also attempted to showcase that the disease predominantly depends on those features that seem meager to us but have noted by doctors as relevant in possibly leading to a higher risk of diseases later. The LR model trained with the dominant features showed an accuracy of 82.92%. For the model forecasting, 0.458 was the probability of class zero and 0.572 for class one, which estimates the probability of a person being diabetic.

Dalakleidi et al. [19] used two datasets named PID, Case 1 and Hippokrateion, which is Case 2 from that the PID is split into 50% for training and 50% for testing, whereas the Hippokrateion has a bifurcation of 70% for training and the other 30% for testing. They used binary logistic regression (BLM), logistic model tree algorithm (LMT), which is a combination of LR and DT learning in simple models. The model’s performance was measured using classification accuracy (ACC) and area under the curve (AUC). BLM achieved an ACC of 80.47 and AUC of 0.85, whereas the LMT achieved an ACC of 77.6 and AUC of 0.84 in Case 1. In Case 2, the BLM outperformed LMT with an ACC of 93.45, whereas the LMT had an ACC of 92.86.

**Fig. 3:**
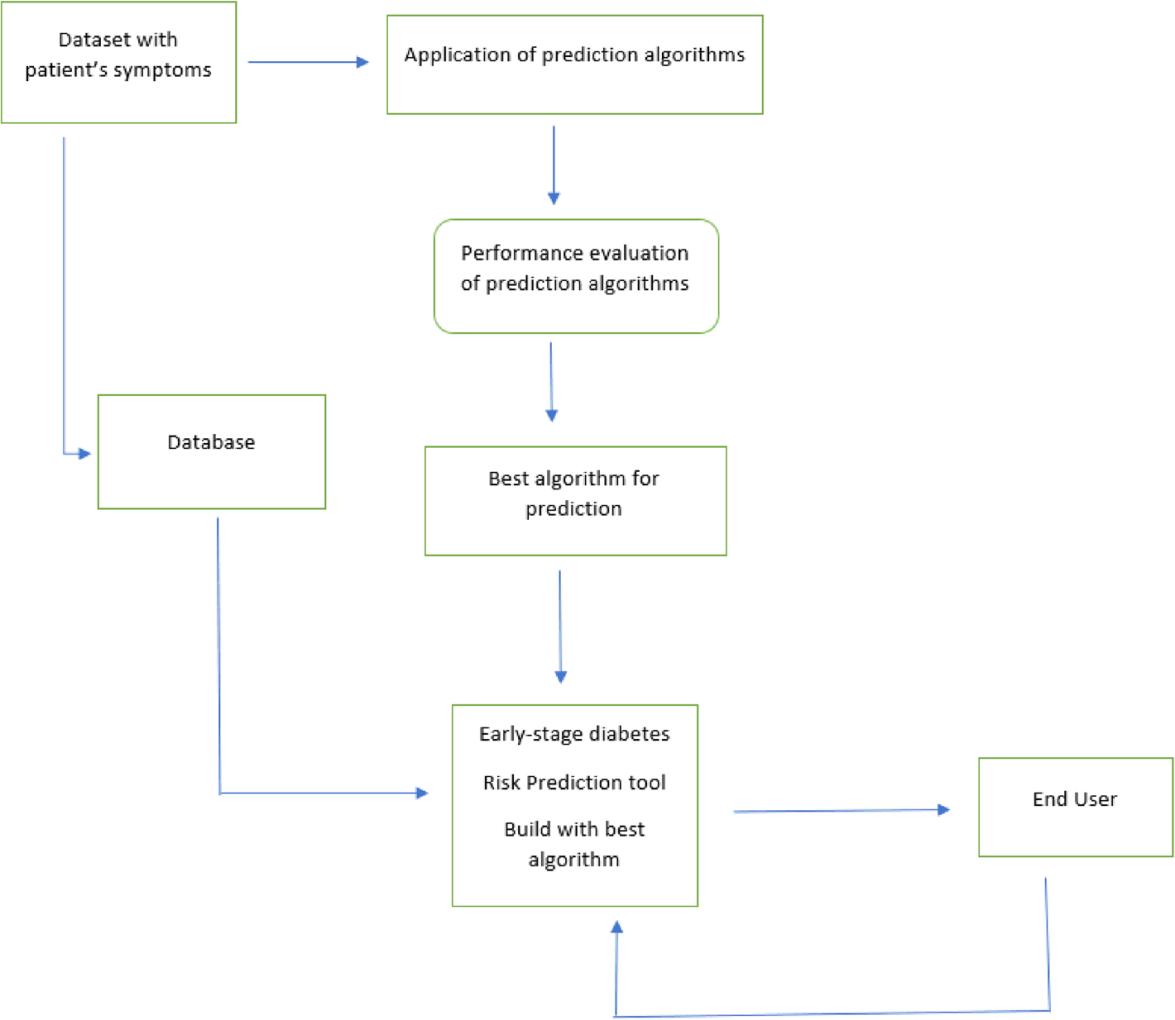
Proposed architecture of the detection system [16]

**Fig. 4:**
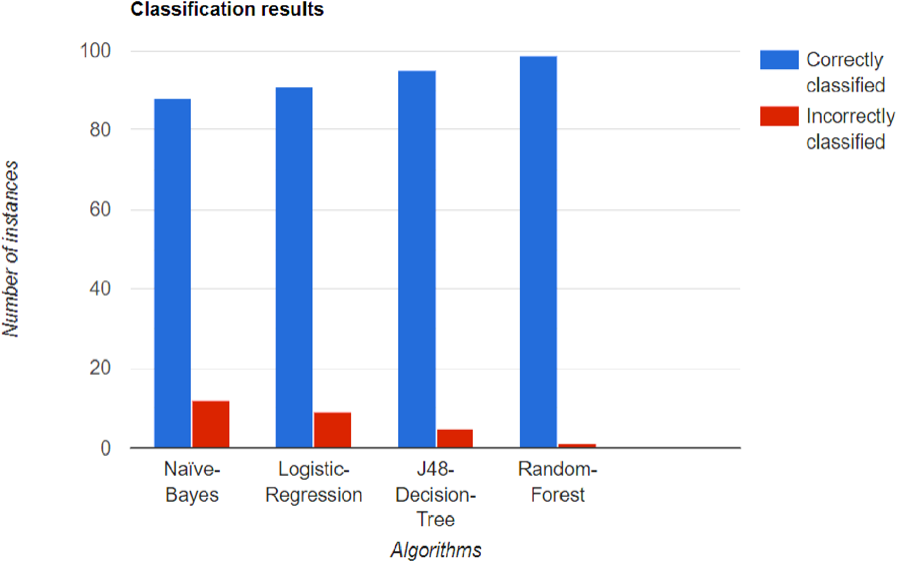
Performance of each algorithm on the newly acquired dataset [20]

Islam et al. [20] used several algorithms to analyze a dataset using the NB and LR algorithms as well as the RF algorithm, after applying 10-fold cross-validation and percentage split evaluation techniques. Figure 3 shows their proposed architecture. The dataset contained records of 520 people who were asked for possible reasons for diabetes. After data pre-processing, there were a total of 314 positive values and 186 negative values. Positive values represent the person being diabetic, and negative implies that they were not. The best result was achieved using the RF algorithm with an accuracy of 99%. Thus, it is an effective algorithm for a newly created dataset. Figure 4 shows exactly how each algorithm performed on modelling and prediction.

**Fig. 5:**
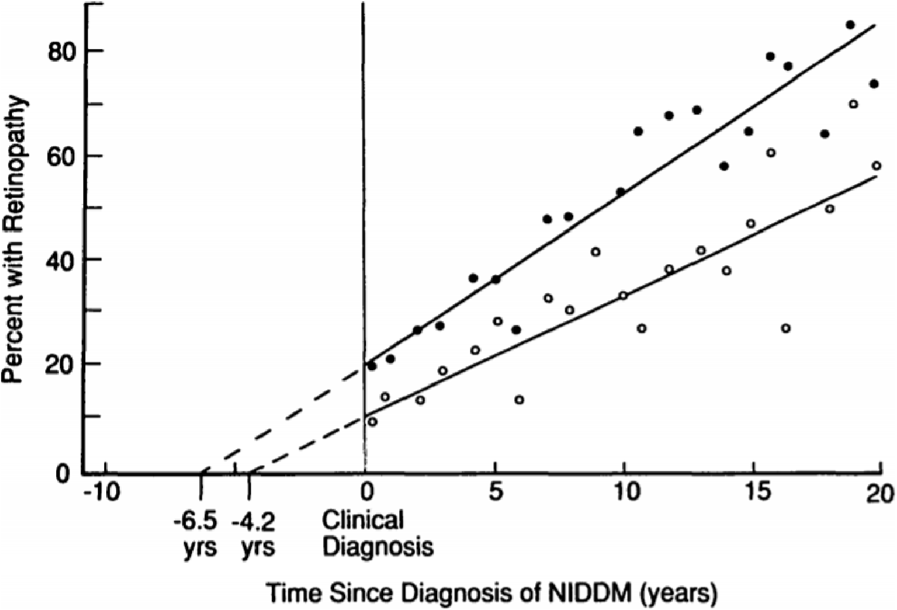
Depicts how at the clinical diagnosis of NIDDM, the patients had the prevalent condition of diabetic retinopathy

Harris et al. [21] performed clinical diagnosis for the detection of non-insulin dependent diabetes mellitus (NIDDM) using weighted linear regression. The relationship between the prevalence of retinopathy and duration of NIDDM was determined according to individual years of duration and assessed using weighted linear regression with weights for each year’s data being inversely proportional to the binomial variance. The author stated that the retinopathy condition is an important parameter for the early diagnosis of the disease. It typically appears almost 4–7 years earlier than the clinical diagnosis of the disease. Figure 5 provides an accurate graph of the obtained results.

Ameena and Ashadevi [22] used the R language to build a model on SVM, DTs, RF, and LR. They used a dataset of 768 women, all of whom were older than 20 years. They used the following features: BMI, blood sugar, number of pregnancies, and diabetes pedigree function. They are defined two classes: 1, which affirmed diabetes and 0 for negation. On a comparison of the accuracies, the author concluded that the RF algorithm showed the maximum correct estimations, with an accuracy of almost 77% compared to the other models.

Daanouni et al. [23] used KNN and the DT algorithm on two datasets, with the first one having 2000 instances and the second having 768. They used eight features or attributes to train the model, such as BMI, glucose, blood sugar, and pregnancy. The authors used 80% for training and the remaining 20% for testing. They used optimized hyperparameters to reduce the loss. The results are plotted on two types of data: pre-processing and without. The comparison of results was performed using a receiver operating curve (ROC). The author concluded that KNN has a maximum accuracy of 97.53% and an AUC of 0.9689. Table 1 shows a comparison table for the accuracies obtained for training the model using the KNN classifier.

**Table 1:** Comparison table obtained for the KNN and the DT model on pre-processed dataset and other without pre-processing [23]

Data	KNN (*k* = 2)	DT
With pre-processing	0.9545	0.936
Without pre-processing	0.9318	0.9434

**Fig. 6:**
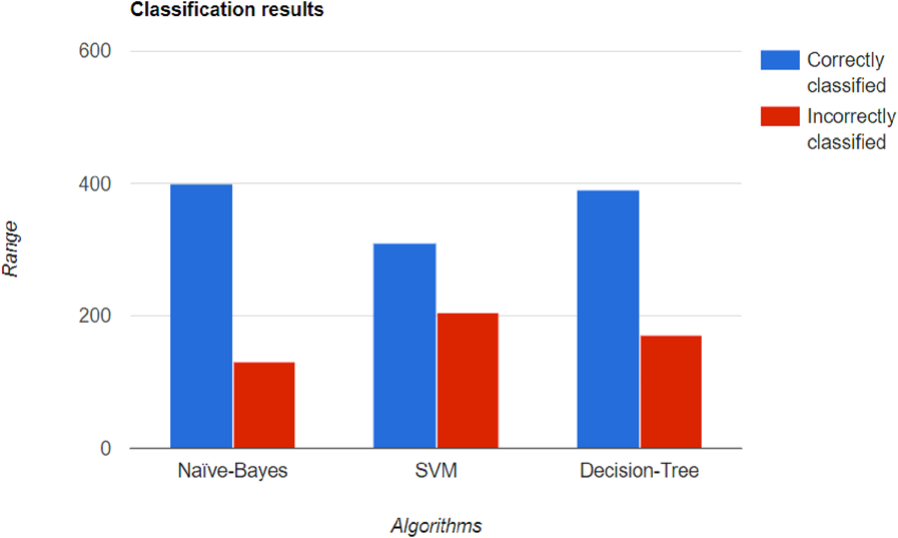
Different classification algorithms result on prediction modelling where NB outgrows the other two

Sisodia D and Sisodia DS [24] used three classifiers, including SVM, NB, and DT. The classification is performed on PIMA Indian diabetes dataset, which is the PIMA Indian diabetes dataset taken from the UCI. To measure the accuracy, internal cross-validation was 10-folds. Accuracy, F-measure, recall, precision, and ROC measures were used. The attributes used were glucose concentration, blood pressure, BMI, age, skin fold thickness, number of pregnancies, 2-h insulin concentration, pedigree function, and class 0 or 1. On modeling, the authors computed that NB showed the maximum accuracy with 586 correctly identifying instances. The following Fig. 6 shows the different types of classifiers used along with number of classified instances.

Ahuja et al. [25] used the dataset from the UCI containing 768 records of women in which 500 were diabetic, while the remaining 268 were not. The authors used eight features for classification and applied a feature selection technique, which is linear discriminant analysis (LDA), to extract the important features required for classification. They used five types of classifiers for machine learning, including SVM, DT, LR, RF, and a multilayer perceptron. The authors used four parameters for evaluation, including accuracy, precision, recall, and F score. Based on these parameters, the authors concluded that multilayer perceptron yields the best results. Table 2 mentions the results using different values of *k*-fold validation.

**Table 2:** Accuracy results of different classifiers at different values of *k*-fold validation (%)

*k*-fold	Support vector classifier	DT	RF	LR	Multi-layer perceptron
*k* = 2	77.6	69	69.9	77.8	77.5
*k* = 4	77.6	69.9	70	77.6	78.7
*k* = 5	77.5	71.5	72.9	77.6	78.2
*k* = 10	77.5	69.5	70	77.6	77.6

**Fig. 7:**
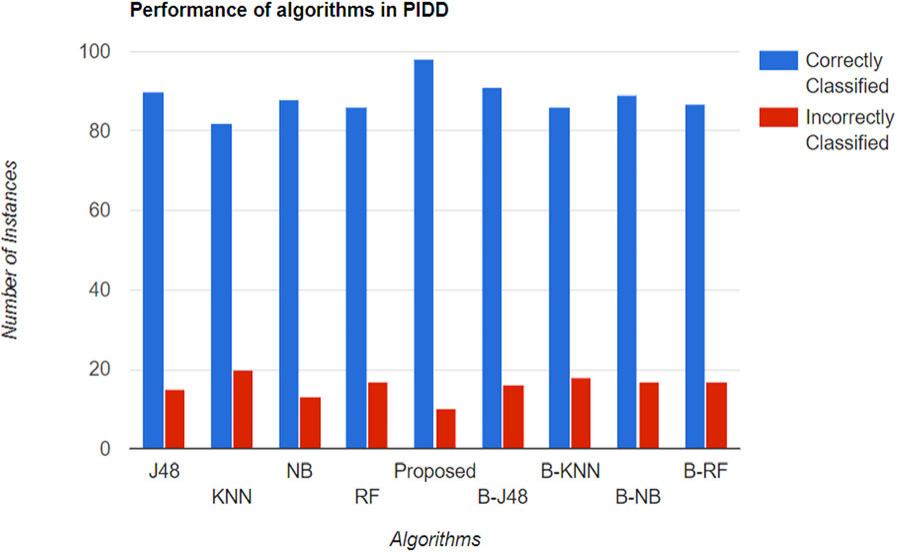
Accuracy of the classification algorithms on the PIMA Indian diabetes dataset. The proposed hybrid model shows the maximum accuracy

Alehegn et al. [26] used the PIMA Indian diabetes dataset with eight features to train on and the 130 D hospital dataset with a larger number of values. There were four classification methods used, including RF, KNN, NB, and J48-DT algorithm. J48 is an upgraded version of the Iterative Dichotomiser 3 (ID3) classification algorithm. A 10 K cross-validation was used for 90% training and 10% testing. The author built a hybrid model consisting of all of the above algorithms. The author concluded that NB and J48 are good for large data computations, and the KNN classifier is better for smaller datasets. Figure 7 shows the different algorithms used along with the correctly and incorrectly identified instances.

Some more work done by other researchers which has been mentioned in Table 3. It contains a study of the machine learning algorithms used in the methods.

**Table 3:** A comprehensive study of the machine learning methods done by some researchers

Algorithm	Method used/innovation	Application and future work	Results and limitations (if specified)	References
J48, AdaBoost, and bagging on base classifier	The model was performed on Canadian Primary Care Sentinel Surveillance Network dataset with several features to train on. The author used ensemble methods AdaBoost on base classifier J48 DT.	The author claimed that these ensemble algorithms can be used on other disease datasets to increase accuracy.	The AdaBoost algorithm with the J48 as the base classifier showed the maximum accuracy followed by bagging and then the J48 classifier. The AROC was used as the parameter.	[27]
NB with clustering	Dataset used was the PIMA Indians Diabetes Dataset with eight attributes. The model is NB performed on prior clustering. This model is compared with only the NB model. Five hundred and thirty-one instances of data were divided into 5 clusters. The fourth cluster was the only one used for testing, which consisted of 148 instances.	By collecting a large amount of data for training, the accuracy can be increased by many-fold, helping people by developing a system that gives them a correct prediction without having to consult a doctor.	The parameters used for evaluation are accuracy, sensitivity, and specificity. The model with clustering showed a 10% increased accuracy, rise in sensitivity by 53.11% but the limitation caused here was the fall of specificity by 10.99% and also a reduced amount of dataset.	[28]
DTs, LR, and NB with bagging and boosting	Initial datasets were collected from primary care units, which (through further changes) consisted of 11 features and a data of 30122 people. The three algorithms are used along with bagging and boosting methods, which are to decrease overfitting and increase accuracy.	The final model obtained with highest accuracy was deployed on a commercial web application.	The following data shows the accuracy with bagging and boosting. DT 85.090, LR 82.308, NB 81.010, Bagging with DT (BG+DT) 85.333, bagging with LR (BG+LR) 82.318, bagging with NB (BG+NB) 80.960, boosting with DT (BT+DT) 84.098, boosting with LR (BT+LR) 82.312, and boosting with NB (BT+NB) 81.019. RF 85.558 shows the maximum accuracy. The ROC was used for final validation.	[29]
LR, KNN, SVM, LDA, NB, DT, and RF	The author collected a raw dataset from Noakhali medical hospital containing 9843 samples with 14 attributes. Eighty percent of the data was taken for training and the rest for testing. All the algorithms chosen by the author were used for model building and then validation was performed on them using *k*-fold.	The author proposed that we can enhance the accuracy of early treatment to lessen the suffering of patients. Additionally, we can implement more classifiers to pick up the leading one for record-breaking performance and extend it to automation analysis.	The RF classifier was the algorithm that performed the best in classifying data and LR showed the worst performance. Although machine learning classifiers are widely used, they still lack in terms of accuracy against deep learning models.	[30]
LR and DTs	The dataset was prepared using a questionnaire carried out for 1487 individuals in which 735 were diabetic and the remaining 752 negatives. A Pearson chi-square test was carried out on all the characteristics. The models’ performance was evaluated on three parameters: accuracy, sensitivity, and specificity. Apart from this, a confusion matrix was also built to determine model performance.	Recently, many researchers have been implementing various algorithms and networks to compare them and find out the most feasible one. DTs and LR are among the ones that are most used.	LR achieved a ACC of 76.54%, sensitivity of 79.4%, and specificity of 73.54% on the testing data while the DT gained an accuracy of 76.97%, sensitivity of 78.11%, and specificity of 75.78%. Overall, the DT model performed better than the LR model. The model poses a limitation of the dataset. It is collected only from one area of China, if it had been collected from different regions, the model implementation could be more practical.	[31]
SVM and LR	Practice fusion de-identified dataset was used for the study taken from Kaggle containing data of approximately 10000 patients. The features were divided into baseline, lab-test, diagnosis, and medication. For the classification task, LR and SVM were deployed. The LR model was implemented using the GLM function and the SVM was used on a linear kernel. The area under the ROC was the parameter used for evaluation.	LR is a model that is widely used in public health and clinical practice for disease detection and to calculate risks.	On using a smaller subset of features, the LR model performed slightly better than the SVM model.	[32]
DTs and NB	The dataset taken for consideration was the PIMA Indian diabetes database. On applying feature selection, the author obtained five features. 10-fold cross-validation was used for data preparation after which the J48 algorithm – DTs and NB is applied. The model performance was evaluated using mean absolute error (MAE), root-mean-square error (RMSE), relative absolute error, root relative squared error, and kappa statistic.	The author proposed to gather information for the dataset from different people to make a more representative model. The work can be further enhanced to include automation.	Using a percentage split of 70:30, the J48 DT algorithm correctly classified 177 instances (76.95%) whereas the NB got an accuracy of 79.56%. The accuracy obtained performed better on the percentage split, which shows the models are not showing good accuracy on larger datasets.	[33]
RF and XG boost	The author used the PIMA diabetes dataset. Using Jupyter Notebook as an IDE, the author trains the model using 8 attributes of the total 9 provided in the dataset. The algorithms used were RF and XGBoost. After setting the hyperparameters the models were trained.	The author suggests the use of more algorithms in this branch of machine learning like hybrid model for better accuracies.	The accuracy gained on the RF classifier came out to be 71.9%. The hybrid model proposed through XG boost gained an accuracy of 74.1%. The accuracies gained on the models were comparatively less compared those already available. The hyperparameter tuning needs to be set better for optimizing the algorithm.	[34]
DT (J48) and NB	The author used the PIMA Indian diabetes dataset with 8 attributes, which was reduced to 5 based on the feature selection. The pre-processing was performed used the WEKA using 10-fold validation. The model was created using the 70% dataset and the rest was used for testing.	In future, it is planned to gather the information from different locales over the world and make a more precise and general prescient model for diabetes conclusion. Future study will likewise focus on gathering information from a later time period and discover new potential prognostic elements to be incorporated. The work can be extended and improved for the automation of diabetes analysis.	The J48 algorithm was 76.95% accurate with other parameters like kappa statistic, MAE, RMSE, relative absolute error, and root relative absolute error. The NB algorithm was accurate up to 79.56%. Since this model is not optimally configured, a developed model would require more training data for creation and testing.	[33]
Genetic algorithms with fuzzy logic	This work is a model implementing the genetic and fuzzy algorithms for effective disease prediction. For the implementation of GA, MATLAB R2006b was used. The principle of feature selection was implemented using fuzzy logic algorithms. Firstly, a simultaneous mapping was performed based on an appropriateness measure of variables values to each class using suitable membership functions according to each type of feature. Then, simple fuzzy reasoning mechanisms were proposed to deal, in unified way, with classification.	The proposed work helps minimize the cost and increase accuracy and can be used in future for better implementations.	Through this approach of GA, the accuracy went up to 87% with the training cost reducing by more than 50%.	[35]
k-NN, NB, DT, RF, SVM LR	These models were created in comparison for detection of type-2 diabetes. A total of 300 samples were taken in which 161 were diabetic, 60 non-diabetic and the rest were unconfirmed. Using feature summarization, eight features were selected with the WEKA tool used. These algorithms were used against a proposed framework that automatically extracts patterns of type 2 DM.	An application of genome wide association and phenome-wide association study in hope for its associations with DM. They proved to be an important association for future models.	Proposed model was evaluated on basis of accuracy, precision, specificity, sensitivity, and AUC. The proposed algorithm gained an AUC of 0.98, which outperforms the state-of-the-art AUC of 0.71.	[36]
LR, DT, RF, SVM	The author used the PIMA Indian women dataset concerned with women’s health with 8 attributes. Different models were trained for this dataset under different hyperparameters.	The author proposed to create advanced models on RF because of its highest accuracy and ability to overcome overfitting.	Different models were compared on basis of accuracy. RF gained the highest accuracy with 77.06% followed by SVM.	[22]
DT – J48, RF	The author obtained dataset from Luzhou from hospital physical examination. An independent test set was taken with 13700 samples. The data contained 14 attributes. Another dataset was the PIMA Indian diabetes dataset. The DT and RF algorithm were implemented in WEKA with principal component analysis.	The author hoped to predict the type of diabetes using a dataset containing the required data which would lead to be an added advantage for improving the accuracy.	The RF and the J48 algorithm achieved an accuracy of 73.95% and 73.88%, respectively, on the Luzhou dataset and 71.44% and 71.67%, respectively, on the PIMA dataset.	[37]
NB and SVM	Patient dataset of 500 records was collected from diabetes healthcare institute who have symptoms of heart disease. The dataset contained 9 attributes. Both the algorithms were implemented on WEKA dataset. For SVM, a radial basis function kernel was used.	Classifiers of this kind can help in early detection of the vulnerability of a diabetic patient to heart disease. There by the patients can be forewarned to change their lifestyle. This will result in preventing diabetic patients from being affected by heart disease, thereby resulting in low mortality rates as well as reduced cost on health for the state.	NB was able to classify 74% of instances correctly. For SVM, the accuracy gained was 95.6%.	[38]
DT, SVM, K-NN RF	The author used the PIMA Indian dataset. Then, data normalization was done followed by feature selection.	The benefit of this optimized machine learning model is that it is suitable for patients with DM and it can be applied to all the fields of health care environments.	The models are compared on the basis of accuracy, sensitivity, and specificity. DT has 78.25% accuracy.	[39]

### Deep learning in diabetes detection

Deep learning is a computational field that is usually involved where high computational power is required. Deep learning focuses on neural networks, their types, training epochs, layers of hidden, input, and output. The input layer is the first layer, and the hidden layers are responsible for all the calculations and manipulations, such as convolutions and pooling. The output layer determines the number of classes for the classification. Because of data augmentation, which means tweaking the data to increase accuracy, is also available in deep learning, it finds many applications with image training. The more layers the network has, the more it is capable of classification. Because of the many advantages, it has been widely used in the medical field to compute results with high accuracy. There are different types of networks with the most proficient artificial neural networks (ANNs), deep neural networks (DNNs), CNNs, and RNNs. Many researchers who work for the detection of a disease compare machine learning and deep learning algorithms to analyze which provides maximum accuracy.

Daanouni et al. [23] used ANNs and DNNs on two datasets of 2000 and 768 instances. They included eight attributes with the label of output as 1 for positive and 0 for negative results. The network was trained on two types of data: pre-processed and non-pre-processed. The DNN model seems to achieve high accuracy on both the data obtained, with an accuracy of 98% for the pre-processed dataset and 99.5% for dataset 1. On dataset 2, the non-pre-processed data had an accuracy of 80.99% and the other 96.35%. Hence, the authors concluded that DNN is an optimal classifier for diabetes detection.

**Fig. 8:**
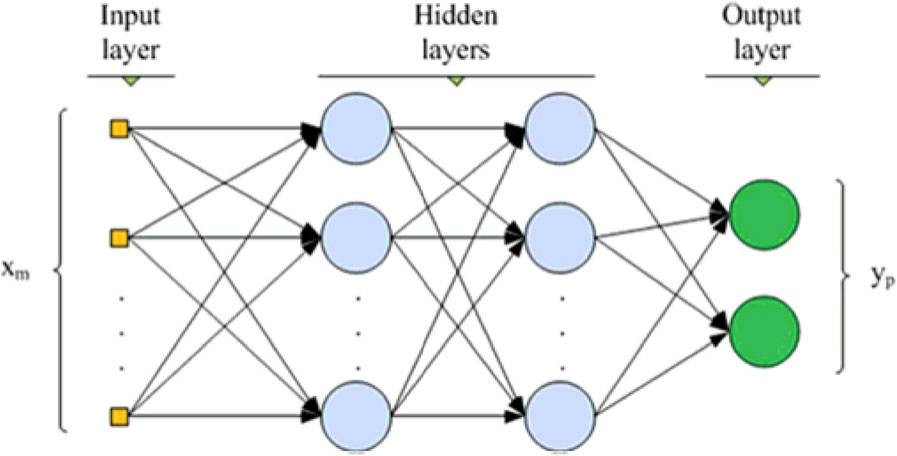
General representation of a neural network, *x*_*m*_ shows the input weights and *y*_*p*_ is the output weights

Rakshit et al. [40] used R, SQL, and Python in a Microsoft Azure machine learning studio environment with the PIMA diabetes dataset, in which 80% was used for training and the other 20% for testing. This dataset is primarily concerned with diabetes in women. This contains eight attributes that are important for model building for a class – 2 neural network. Figure 8 shows the general representation of the neural network. The hidden layer had 100 nodes, with the output layer connected to the nth hidden layer. With the model trained for over 1000 epochs with a learning rate of 0.01, they achieved an accuracy of 83.3% on a dataset with 262 negative cases and 131 positive cases.

Sapon et al. [41] presented diabetes prediction using supervised ANNs. The dataset comprises approximately 250 patients with 27 variables or features,, where exactly 50% was used for training, while the remaining 50% was used for testing with the MATLAB tool. The gradient algorithms used included the Fletcher-Powell conjugate gradient, Polak-Ribiére conjugate gradient, and scaled conjugate gradient. These algorithms were used to train the model and then analyzed using the correlation coefficient (CC) R. The results of these algorithms are plotted at different epochs against the mean square error. Based on a comparison of the values of R, the authors conclude that the scaled conjugate gradient confirms the highest accuracy with a value of 0.88, followed by the Fletcher-Powell conjugate gradient with a value of 0.097219 and, finally, the Polak-Ribiére conjugate gradient with 0.056466.

Refs. [31, 42] both performed detection using an ANN. Ref. [42] used the PIMA Indian population dataset for women in Phoenix, while [31] performed this algorithm on a questionnaire model that contained data of 1487 people with positive and negative results. The features that remained common for modeling were BMI, age, weight, marital status, pregnancies, and ref. [31] collected a large number of variables, such as consumption of alcohol, meat, cigarette smoking count, beverage variety, and their counts and routine for exercise and sleep. Considering the structure of the ANN, ref. [31] had approximately 15 hidden nodes, while the same varied from 0 to 5 in ref. [42]. Ref. [31] achieved a ACC of 73.52% against ref. [42], who achieved an accuracy of 80.21% on the test data.

Ref. [43] conducted a comparative study of neural networks in diabetes detection. Using the PIMA Indian diabetes set again, they used the eight common features required for model preparation. With the first 576 cases used for testing, they used a 10-fold cross-validation technique too estimate the results. The author used a multilayer neural network (MLNN) with 50 neurons for each hidden layer and an output layer using the non-sigmoid activation function and a PNN, which is a probabilistic neural network with a single hidden layer. The author concluded his results on accuracy, showing that the MLNN model achieved an accuracy of 79.62, and the PNN achieved an accuracy of 78.65.

**Fig. 9:**
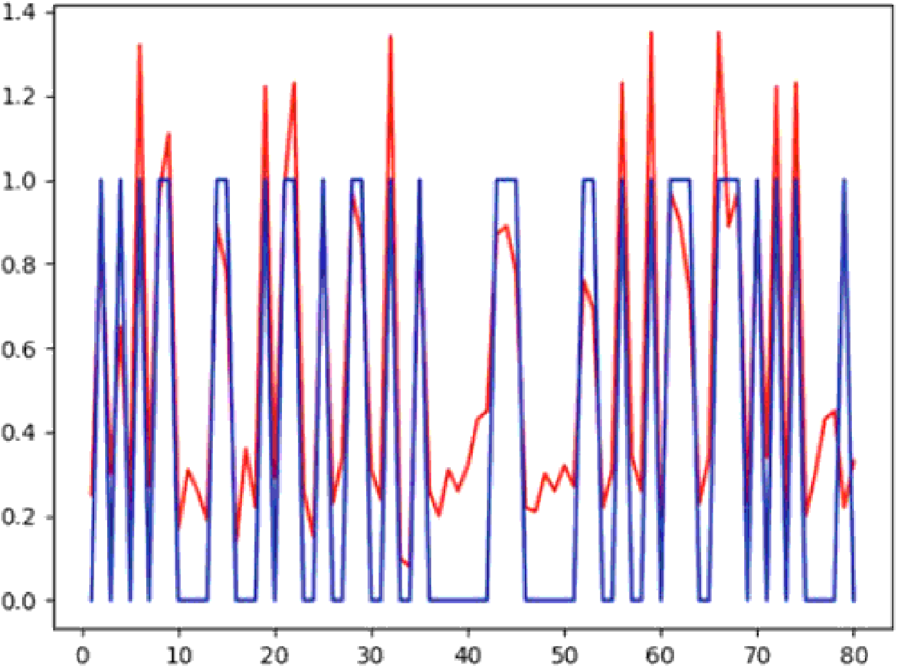
The graphs plotted between actual value shown by the blue line and predicted values shown by the red line

Ref. [44] used the PIMA diabetes set and computed the model using an ANN and used eight features for modeling. The author explained the different functions used for pre-processing and model training. The activation function was a sigmoid function, and backpropagation is used to calculate the gradient of the loss function. The error function computed the final error to be approximately 8% at the end of the model building. The results were validated on ROC and RMSE. The author achieved an ROC area of 0.88 and RMSE equal to 0.39, which is a FAIR classifier. Figure 9 shows the results as plotted in form of lines.

Ref. [29] used an ANN for his model trained over a dataset consisting of over 30000 instances and 11 features to train on. With the hidden layers equal to 12, the values of the layers were calculated using the sigmoid function. Bagging and boosting methods were implemented. Bagging was also set to reduce the variance in the model, and boosting was performed to reduce the error. The neural network with bagging achieved an accuracy of 85.324%, followed by the ANN model with boosting with an accuracy of 84.815%, and then the ANN with 84.532%. Finally, the author validates the final comparison using an ROC.

Ref. [45] used machine learning and deep learning techniques to detect DM. The dataset used here was the PIMA Indian diabetes dataset consisting of 768 features and eight features to train on with a total of 500 instances belonging to the non-diabetic class and the remaining 268, which are diabetic class. Sixty percent of the data was selected for training and the remaining 40% for testing; a CNN was used. They are composed of three layers, with the classification is performed by the output layer. The prediction accuracy achieved using the model was 76.81%.

Some more work done by other researchers which has been mentioned in Table 4. It contains a study of the deep learning algorithms used in the methods.

**Table 4:** A comprehensive study of the deep learning methods done by some researchers

Algorithm	Method used/innovation	Application	Results and limitations (if specified)	References
Deep belief neural network	The dataset used was PIMA Indian diabetes dataset with 768 instances and 8 features. The activation hidden function used was ReLU rectifier linear unit with three hidden layers and sigmoid as the input activation function. The batch size at 100 and epochs set to 5.	As the author compares his network with conventional methods of machine learning classifiers and it obtained high results, they believe that this network can be tweaked according to convenience and used for detection of other diseases as well.	The results of the network proposed by the author and conventional methods were seen on three parameters: recall value, precision and F1 measure. This network obtained high recall, precision, and F1 measure values, which shows that it is a good model.	[46]
Long short-term memory (LSTM) neural networks – RNNs	The author used Direct Net Inpatient Accuracy Study dataset which contains approximately 110 instances. The neural network model proposed here has one LSTM layer. The model works on predicting blood sugar levels. For the search grid, the author takes into consideration LSTM units, Dense units, and sequence lengths. The parameter taken for evaluating model performance is RMSE.	The author elaborated on the real-life applications by proposing to deploy LSTMs models on mobile platforms, apps, and cloud servers for availability to the masses.	It was concluded that the use of LSTM for blood glucose level prediction is promising. The values of RMSE obtained are 4.67 which is the minimum and 29.12 being the max value. Missing data is an issue for the model as if the patient removes the CGM device, the model should be trained in a format that it can automatically handle the missing data.	[47]
Deep prediction model	The data taken was of six individuals in the age group 22-29 years. Deep prediction model is a multiple layer model consisting of data driven predictors which is fed with glucose measurement and time series. The first part is the autoregressive models with external inputs or the ANN’s followed by the extreme learning machine (ELM) and the glucose level predictions are given. The learning speed of ELM’s is very fast and also, they are easier to implement.	ELM models are easy to implement any classification application and can do approximations for continuous functions; they are widely used in this field of research for implementing efficient models.	The model was evaluated on three parameters–RMSE, CC, and time lag (TL) at different glucose concentrations for PH = 15, 30, and 45 mins with input combinations of three types involving glucose and sugar concentrations. For all PHs the linear models along with the ELM outperforms the ANN-ELM achieving reduced TL, reduced RMSE and increased CC.	[48]
Empirical mode decomposition (EDM) and LSTM	The dataset was obtained from shanghai hospital containing 174 instances. The data is used for training two models: one is LSTM and the other is LSTM+EDM. LSTMs are improved versions of RNN and EDM is an adaptive signal decomposition method for non-linear and non-stationary methods. The performance evaluation measures are MAE and RMSE.	The author proposed a more accurate model by treating it with real-time data or personalized data to give better results. Deploying of the model to mobile clients was also suggested.	The parameters considered for evaluating the performance accuracy were the MAE and RMSE. The MAE measures the prediction error and the RMSE gives the deviation between the observed value and the truth value. The results are observed over a time interval of 30, 60, 90 and 120 mins.	[49]
Temporal convolutional network with vanilla LSTM	The raw dataset included two male instances and four female instances. Feature selection was performed on the dataset followed by the hyperparameter tuning. The LSTM network used had 3 hidden layers with size equals 50. The TCN block has 10 layers with each having a dilation factor of 2 and kernel size 4.	The author stated that this study of using DL algorithms in time-series prediction would help practitioners and researchers for selecting appropriate models with pragmatical parameters.	The author used three parameters for evaluation, namely RMSE, temporal gain, and normalized energy of second order. The values of RMSE, temporal gain, and energy were much higher for the vanilla LSTM than the TCN. The limitation for the model was that it does not collect data that involves deeper meaning for personalized output.	[50]
ANN	Medical dataset taken from Noakhali medical college containing data of 9483 patients with 14 features. The dataset was split into 80% for training and the rest 20% for testing. For ANN, the author chose the SoftMax activation function with six hidden layers. The training was done using ReLU activation function with 25 epochs.	Because ANNs achieve high accuracy, the author recommends that using those for model prediction and detection of diseases would be a much better alternative than other ML models.	For increasing model performance, an extra hidden layer was added and the epochs were increased to 100. The accuracy came out to be 95.14% on the testing data and 96.42% on training. The author claims that models achieved low accuracy because smaller datasets and models incapability of adaption to various datasets.	[30]
LSTM and Bi-LSTM (RNN)	The author collected the dataset from real patients by monitoring their health. The model was trained and tested on 26 datasets containing real-time CGM data. The model consists of one LSTM layer and one Bi-LSTM layer each with 4 units, and three fully connected layers with 8, 64 and 8 units, respectively. The epochs ranged from 100 to 2000. The parameters used for evaluation were RMSE, CC, and TL.	The author proposed the model use for oral drugs, insulin pens, and the CSII pumps, which all incorporate CGM measurements.	The results were calculated at different PH = 15, 30, 45, and 60 mins. On running the models for different epochs from 100 to 2000 on a difference of 100, epoch number 900, 1300, 1500, 1700 were the ones that showed good accuracy and hence they were chosen as pre-train epoch number for particular PH levels.	[51]
CNN	A risk prediction can accurately identify the risk of the disease using CNN. The study was based on a group of steel workers numbered around 5900. A research survey was conducted to gain real-time data on features like gender, age, disease history, lifestyle, and physical examinations. The model was evaluated using ROCs and area under curve.	It provides a basis for self-health management of steel workers, facilitate the rational allocation of medical and health resources and the development of health services, and provide a basis for government departments to make decisions.	The prediction accuracy of the model in the three data sets was 94.5%, 91.0%, and 89.0%, respectively, and the AUC was 0.950 (95%CI: 0.938–0.962), 0.916 (95%CI: 0.888–0.945), and 0.899 (95%CI: 0.859–0.939). It shows that the established model can accurately predict the risk of type-2 diabetes in steel workers.	[52]
AlexNet and GoogLeNet	In this study, the author developed a model called IGRNet to detect and diagnose prediabetes effectively using a 12-lead electrocardiogram lasting 5 s of 2251 case data. The neural networks used were compared with traditional ML algorithms like SVM, RF, and KNN.	The author suggested to use this hybrid model for future predictions due to its efficient performance.	The results of the networks were compared over different activation functions. The IGRNet model gained an accuracy of 85.6%, which was higher than any other model used for comparison.	[53]
DNN	The data collection was retrieved from the UCI machine learning repository – PIMA Indian diabetes dataset. For the neural network, the hidden layer count is 4. The numbers of neurons in the layers were 12, 16, 16, and 14.	The proposed system will be supportive for the medical staff and as well as for the common people because with five-fold cross-validation, the accuracy was more than any other model and on comparing it with other established models of authors, it came out to be the highest.	The data samples were divided into 5-fold and 10-fold cross-validation. The accuracy gained on this model for five-fold was 98.04% and for ten-fold was 97.27%. The results were also analyzed through ROCs and F1 score.	[54]
LSTM and GRU	The dataset used included records of over 14000 patients from 2010 to 2015. Episodes were used to represent those measures. Each sequence had 30 features. The dataset used for training the LSTM and GRU models. The results were compared with the MLP models.	The models received a high accuracy of 97% even with 3-d length sequence.	The models, LSTM and GRU achieved higher than MLP. For longer dependencies, LSTM outperformed while on shorter sequences, GLU performed better. They gained an accuracy of over 97%. Due to lack of datasets that are more concentrated toward type-2 diabetes, replicating this work using different datasets can be difficult.	[55]
Multilayer perceptron network	The author used the PIMA Indian diabetes dataset. The ANN developed contains 4 layers for the MLP network having 8-12-8-1 nodes. Three more networks were formed with nodes 8-32-32-1, 8-64-64-1 and 8-128-128-1. With the ReLU activation function in the input layer and hidden layers, the output layer is sigmoid.	The author proposed to extend this work for further accuracy increase to help in early prediction for diabetes.	The results for the perceptron were calculated for 10 runs for 150 epochs. The highest average accuracy was gained by the network having nodes 8-128-128-1 followed by 8-64-64-1 and then 8-32-32-1.	[56]
ELM algorithm	The data was acquired from multiple sources, including medical laboratories, hospitals, and public datasets. The dataset was pre-processed and meant to include 12 important attributes affecting diabetes. ELM has faster ability for training. Three hundred and twenty samples were used for training and 480 for training.	The goal of this research was the study of diabetic treatment in healthcare using big data and machine learning. It presents a big data processing system by employing an ELM algorithm.	The proposed approach was proven to be efficient. The goal was to reduce the FP and FN and boost the precision and recall rates, which was achieved by the author.	[57]
Neural network	The author obtained dataset from Luzhou from hospital physical examination. An independent test set was taken out with 13700 samples. The data contained 14 attributes. Another dataset was the PIMA Indian diabetes dataset. This method is a two-layer network with sigmoid hidden and SoftMax output neurons.	The author hopes to predict the type of diabetes using a dataset which is a lead for improving accuracy.	The neural network gained an accuracy of 74.14% on Luzhou dataset and 74.75% on PIMA Indian dataset with the use of principle component analysis.	[37]
CNN	Dataset used by the author was taken from National Institute of Diabetes, which consists of nine parameters. Fuzzification was done on the dataset for CNN which becomes populated. The α values of the neural network were 2 and 5. These networks were compared with a CNN.	Fuzzification proves to be of a useful nature since it provides diverse data to train on.	The neural network with value of α = 2 performed better α value 5. CNN performed better than both of them.	[58]

### Challenges and future scope

Although there many methods and algorithms which have been proposed in the field of machine learning or deep learning, many challenges remain, as mentioned by the authors in their works. The first concern that comes to mind when building a model is data. Refs. [32, 33, 49] were some of the authors that referenced the problem. One of the biggest problems encountered during the survey of the papers was finding articles and papers that did not relate to the popular PIMA Indian dataset. This dataset was chosen to ensure that the models provided good results because of the establishment of the dataset. The datasets are either too small or inadequate, or they lack real-time data. Small datasets pose a problem of overfitting on the model, which shows higher accuracy, but they are not able to deal with newer testing data. Hence, the model is not feasible for real-time implementation. Some authors dealt with CGM data, which was real-time, but the model training with that data was not efficient. The datasets were also selected from particular regions that are not representative of a common system. Different regions have different people and lifestyles. Generally, researchers spend 80% of their time cleaning and managing data for model training. Hence, data complexity leads to higher cost and maintenance charges. The next step is feature selection. While some authors neglected some of the features, some grouped them for feasible training. Every dataset poses the problem of having appropriate features to cater to the needs of a single algorithm. After all the data are made available, the technical stacks are finalized. Many tools are available to construct machine learning models, but choosing a model to optimize performance is also necessary. The next challenge is debugging. This becomes easy if tools such as Jupyter Notebooks are used where the code is divided into cells. This becomes difficult when the model runs on automation batch processes. In addition, as there are only a few diabetes datasets available on the Internet; more public data should be available for research. More research should be performed using heart rate, as it requires less bandwidth, and its computational complexity is also low. They can also be used in cloud or mobile devices. HR signals should also be used to detect other cardiac diseases. In some cases, authors required a time-series dataset. Since they are not available across any online resource, it is difficult to replicate such work. Such special models require extensive tuning and large datasets for both training and testing.

The next part is the construction of an actual model. To achieve perfect accuracy, many parameters must be adjusted. Random states, kernel, number of trees, hyperparameter tuning, and various others are considered while creating a model. Selecting a correct algorithm with suitable hyperparameters should also be performed precisely. Some classification models will only train on a single parameter, which results in a decreased accuracy for the model in real-time detection. It is evident from the analysis of these schemes in all classes that most of them suffer from either a single data input parameter or the feature selection is not optimal. Along with such restrictions on parameters, few classification-based schemes are purely dependent on kinds of hardware devices, which increases the difficulty of availability and adaptability of these schemes.

A healthcare-based machine learning model is only useful if it can be used for the benefit of people. Here, the model deployment in practical applications is critical. Many authors have proposed deploying models on mobile platforms. In real-life implementations, only engineers with background and experience with cloud servers and DevOps can deploy models. In this ongoing process, many issues need to be considered, such as how frequently the predictions are required to be displayed or the number of applications that are required for model processing. Although considerable precautions were taken to ensure there were no discrepancies in the study, no study could claim to be perfect and there is always scope for improvement. The development of more inquisitive study providing deeper insights into aspects that enable the predictive power of models rather than only pre-defined parameters such as accuracy, precision, F1 score, ROC, and AUC would be beneficial in the future. For classification, to distinguish between diabetic and normal profiles, clustering-based schemes provide accurate results. However, most of the clustering algorithms struggle with plug-n-play problems, which means that they usually contain human intervention during classification and analysis, which involves the possibility of error.

Considering all the above challenges, it can still be considered that they can be overcome in the future. Scholars and clinicians will continue to work toward the construction of larger and better datasets and design more efficient models and algorithms for better classification and accuracy. Any of the diseases occurring on a wide scale, such as diabetes, can be controlled through artificial intelligence techniques and automation. One can create state-of-the-art efficient models based on studies that provide early detection of diabetes and can help people to further change their lifestyle. Because deep learning performs better on most datasets, it should be combined with different algorithms to achieve better accuracy and performance. Hybrid schemes play an important role in improving the performance of the models. Through early detection, patients can be treated much earlier to avoid further risks of heart problems in cases of diabetes. Any model that can be deployed on mobile platforms should cater to the masses for their help and be representative. An implication of this survey is that ML models that have yielded efficient results that can be utilized by future researchers to further polish and improve as well as create a pipeline or an ensemble of correct and efficient models to increase the chances of predicting the disease with even more probability. Such models can be further improvised to automate the system created so that it can deal with newer data without problems.

## Conclusions

Diabetes can be devastating after a certain period if not detected or diagnosed correctly. Many machine learning methods have been discussed, starting from different basic algorithms such as the LR, SVM, DTs, to further classification including the ID3, C4.5, C5.0, J48 and CART and NB. Ensemble methods, such as bagging, boosting, and RF regressors, are further used to enhance the accuracy and performance of models. These techniques have been implemented on all types of platforms such as Python or MATLAB, and the models have been analyzed using different parameters such as area under curve or confusion matrices or mathematical terms such as the RMSE or MAE. Machine learning has been introduced in medical diagnosis systems as it has proven to be accurate in detection, successful in application to treatments, and is more cost effective. Although the above are very strong classifiers, we believe that deep learning, which is a subset of machine learning, can learn large amounts of unstructured and unlabeled data. Deep learning models are more complex and accurate. Different models for deep learning start from the most basic ANNs to convolutional nets to further RNNs, including LSTM and Bi-LSTM. Temporal and deep belief networks have also been discussed. In contrast, deep learning involves some shortcomings such as increased computational time, resources, and frequent adjustment of the parameters. Deep learning performs better on image datasets; therefore, for diabetes diagnosis, images would be better. Most researchers have implemented several algorithms in both machine and deep learning to compare their performance on the data, while others have combined two or three methods to gain more accuracy on a single system.

Researchers, clinical practitioners, and people in the industry widely believe that artificial intelligence has the power to alter the ongoing situations of late medication and detection due to human errors. Automation has the capability to construct efficient and reliable medical detection systems. Machine learning, by means of its powerful predictive and classification models, plays an important role in helping to achieve this.

## Data Availability

All relevant data and material are presented in the main paper.

## Abbreviations

OPTICS: Ordering points to identify cluster structure
DBSCAN: Density based spatial clustering of applications
DM: Diabetes mellitus
BIRCH: Balanced iterative reducing and clustering algorithm using hierarchies
LSTM: Long short-term memory
ID3: Iterative Dichotomiser 3
SVM: Support vector machine
KNN: K-nearest neighbors
TP: True positive
TN: True negative
FP: False positive
FN: False negative
BLM: Binary logistic regression
LMT: Logistic model tree algorithm
ACC: The classification accuracy
AUC: Area under the curve
LDA: Linear discriminant analysis
ANN: Artificial neural network
RNN: Recurrent neural network
CNN: Convolutional neural network
DNN: Deep neural network
MAE: Mean absolute error
CC: Correlation coefficient
TL: Time lag
ROC: Receiver operating curve
ELM: Extreme learning machine
NIDDM: Non-insulin dependent diabetes mellitus
BMI: Body mass index
DT: Decision tree
LR: Logistic regression
RF: Random forest
NB: Naïve Bayes
MLNN: Multilayer neural network
RMSE: Root-mean-square error
EDM: Empirical mode decomposition

## References

[1] Nakahara T, Hyogo H, Yoneda M, Sumida Y, Sumida Y, Fujii H (2013). Type 2 diabetes mellitus is associated with the fibrosis severity in patients with nonalcoholic fatty liver disease in a large retrospective cohort of Japanese patients. J Gastroenterol.

[2] Solanki P, Baldaniya D, Jogani D, Chaudhary B, Shah M, Kshirsagar A (2021). Artificial intelligence: new age of transformation in petroleum upstream. Pet Res (in press).

[3] Duplaga M (2004) The impact of information technology on quality of healthcare services. In: Bubak M, van Albada GD, Sloot PMA, Dongarra J (eds) Computational science - ICCS 2004. 4th international conference, Kraków, Poland, June 2004. Lecture notes in computer science, vol 3039. Springer, Berlin, Heidelberg, pp 1118-1125. 10.1007/978-3-540-25944-2_145

[4] Lassi M, Sonnenwald DH (2010) Identifying factors that may impact the adoption and use of a social science collaboratory: a synthesis of previous research.Inf Res15(3)

[5] Bonfiglio S (2012) The role of ICT in a healthcare moving from “clinical-centric” to “patient-centric”. In: Donnelly M, Paggetti C, Nugent C, Mokhtari M (eds) Impact analysis of solutions for chronic disease prevention and management. 10th international conference on smart homes and health telematics, June 2012. Lecture notes in computer science, vol 7251. Springer, Berlin, Heidelberg, pp 250-253. 10.1007/978-3-642-30779-9_37

[6] Poston RS, Reynolds RB, Gillenson ML (2006). Technology solutions for improving accuracy and availability of healthcare records. Inf Syst Manag.

[7] Duan L, Street WN, Xu E (2011). Healthcare information systems: data mining methods in the creation of a clinical recommender system. Enterp Inf Syst.

[8] Saiti K, Macaš M, Štechová K, Pithová P, Lhotská L (2017) A review of model prediction in diabetes and of designing glucose regulators based on model predictive control for the artificial pancreas. In: Bursa M, Holzinger A, Renda ME, Khuri S (eds) Information technology in bio- and medical informatics. 8th international conference ITBAM 2017, August 2017. Lecture notes in computer science, vol 10443. Springer, Cham, pp 11-19. 10.1007/978-3-319-64265-9_6

[9] Haritha R, Sureshbabu D, Sammulal P (2019) Diabetes detection using principal component analysis and neural networks. In: Santosh KC, Hegadi RS (eds) Recent trends in image processing and pattern recognition. Second international conference, RTIP2R 2018, December 2018. Communications in computer and information science, vol 1036. Springer, Singapore. 10.1007/978-981-13-9184-2_24

[10] Chen Q, Alrowais R, Burhan M, Ybyraiymkul D, Shahzad MW, Li Y (2020). A self-sustainable solar desalination system using direct spray technology. Energy.

[11] Kunekar PR, Gupta M, Agarwal B (2019) Detection and analysis of life style based diseases in early phase of life: a survey. In: Somani AK, Ramakrishna S, Chaudhary A, Choudhary C, Agarwal B (eds) Emerging technologies in computer engineering: microservices in big data analytics. Second international conference ICETCE 2019, February 2019. Communications in computer and information science, vol 985. Springer, Singapore. 10.1007/978-981-13-8300-7_6

[12] Cahn A, Shoshan A, Sagiv T, Yesharim R, Goshen R, Shalev V (2020). Prediction of progression from pre-diabetes to diabetes: development and validation of a machine learning model. Diabetes Metab Res Rev.

[13] Thenappan S, Rajkumar MV, Manoharan PS (2020) Predicting diabetes mellitus using modified support vector machine with cloud security. IETE J Res. 10.1080/03772063.2020.1782781. (in press)

[14] Bai BGM, Nalini BM, Majumdar J, Shetty NR, Patnaik LM, Nagaraj HC, Hamsavath PN, Nalini N (2019). Analysis and detection of diabetes using data mining techniques-a big data application in health care. Emerging research in computing, information, communication and applications.

[15] Shu T, Zhang B, Tang YY, Chengdu IEEE (2018) 15-18 July 2018. 10.1109/ICWAPR.2018.8521271

[16] Islam MT, Raihan M, Farzana F, Aktar N, Ghosh P, Kabiraj S (2020) Typical and non-typical diabetes disease prediction using random forest algorithm. In: Abstracts of the 11th international conference on computing, communication and networking technologies, IEEE, Kharagpur, 1-3 July 2020. 10.1109/ICCCNT49239.2020.9225430

[17] Choudhury A, Gupta D (2019) A survey on medical diagnosis of diabetes using machine learning techniques. In: Kalita J, Balas VE, Borah S, Pradhan R (eds) Recent developments in machine learning and data analytics. Advances in intelligent systems and computing, vol 740. Springer, Singapore, pp 67–78. 10.1007/978-981-13-1280-9_6

[18] Shukla AK, Pant M, Sharma TK, Arya R, Sahana BC, Zolfagharinia H (2020). Patient diabetes forecasting based on machine learning approach. Soft computing: theories and applications. Advances in intelligent systems and computing.

[19] Dalakleidi KV, Zarkogianni K, Karamanos VG, Thanopoulou AC, Nikita KS (2013) A hybrid genetic algorithm for the selection of the critical features for risk prediction of cardiovascular complications in Type 2 Diabetes patients. In: Abstracts of the 13th IEEE international conference on BioInformatics and BioEngineering, Chania, 10-13 November 2013. 10.1109/BIBE.2013.6701620

[20] Islam MMF, Ferdousi R, Rahman S, Bushra HY (2020) Likelihood prediction of diabetes at early stage using data mining techniques. In: Gupta M, Konar D, Bhattacharyya S, Biswas S (eds) Computer vision and machine intelligence in medical image analysis. Advances in intelligent systems and computing, vol 992. Springer, Singapore, pp 113–125. 10.1007/978-981-13-8798-2_12

[21] Harris MI, Klein R, Welborn TA, Knuiman MW (1992). Onset of NIDDM occurs at least 4-7 yr before clinical diagnosis. Diabetes Care.

[22] Ameena RR, Ashadevi B (2020) Predictive analysis of diabetic women patients using R. In: Peter JD, Fernandes SL (eds) Systems simulation and modeling for cloud computing and big data applications. Elsevier Inc., Amsterdam. 10.1016/B978-0-12-819779-0.00006-X

[23] Daanouni O, Cherradi B, Tmiri A (2019) Predicting diabetes diseases using mixed data and supervised machine learning algorithms. In: Abstracts of the 4th international conference on smart city applications, ACM, Casablanca, 2-4 October 2019. 10.1145/3368756.3369072

[24] Sisodia D, Sisodia DS (2018). Prediction of diabetes using classification algorithms. Procedia Comput Sci.

[25] Ahuja R, Sharma SC, Ali M (2019). A diabetic disease prediction model based on classification algorithms. Ann Emerg Technol Comput.

[26] Alehegn M, Joshi RR, Mulay P (2019). Diabetes analysis and prediction using random forest, KNN, Naïve Bayes, and J48: an ensemble approach. Int J Sci Technol Res.

[27] Perveen S, Shahbaz M, Guergachi A, Keshavjee K (2016). Performance analysis of data mining classification techniques to predict diabetes. Procedia Comput Sci.

[28] Khan NS, Muaz MH, Kabir A, Islam MN (2019). A machine learning-based intelligent system for predicting diabetes. Int J Big Data Anal Healthc.

[29] Nai-Arun N, Moungmai R (2015). Comparison of classifiers for the risk of diabetes prediction. Procedia Comput Sci.

[30] Kocher T, Holtfreter B, Petersmann A, Eickholz P, Hoffmann T, Kaner D (2019). Effect of periodontal treatment on HbA1c among patients with prediabetes. J Dent Res.

[31] Meng XH, Huang YX, Rao DP, Zhang Q, Liu Q (2013). Comparison of three data mining models for predicting diabetes or prediabetes by risk factors. Kaohsiung J Med Sci.

[32] Sheikhi G, Altınçay H (2016) The cost of type II diabetes mellitus: a machine learning perspective. In: Kyriacou E, Christofides S, Pattichis CS (eds) XIV mediterranean conference on medical and biological engineering and computing 2016. IFMBE proceedings, vol 57. Springer, Cham, pp 818-821. 10.1007/978-3-319-32703-7_160

[33] Iyer A, Jeyalatha S, Sumbaly R (2015). Diagnosis of diabetes using classification mining techniques. Int J Data Min Knowl Manag Process.

[34] Barik S, Mohanty S, Mohanty S, Singh D (2021) Analysis of prediction accuracy of diabetes using classifier and hybrid machine learning techniques. In: Mishra D, Buyya R, Mohapatra P, Patnaik S (eds) Intelligent and cloud computing. Smart innovation, systems and technologies, vol 153. Springer, Singapore, pp 399–409. 10.1007/978-981-15-6202-0_41

[35] Ephzibah EP (2011) A hybrid genetic-fuzzy expert system for effective heart disease diagnosis. In: Wyld DC, Wozniak M, Chaki N, Meghanathan N, Nagamalai D (eds) Advances in computing and information technology. first international conference, ACITY 2011, July 2011. Communications in computer and information science, vol 198. Springer, Berlin, Heidelberg, pp 115-121. 10.1007/978-3-642-22555-0_13

[36] Zheng T, Xie W, Xu LL, He XY, Zhang Y, You MR (2017). A machine learning-based framework to identify type 2 diabetes through electronic health records. Int J Med Inform.

[37] Zou Q, Qu KY, Luo YM, Yin DH, Ju Y, Tang H (2018). Predicting diabetes mellitus with machine learning techniques. Front Genet.

[38] Parthiban G, Srivatsa SK (2012). Applying machine learning methods in diagnosing heart disease for diabetic patients. Int J Appl Inf Syst.

[39] Challa M, Chinnaiyan R (2019) Optimized machine learning approach for the prediction of diabetes-mellitus. In: Smys S, Tavares JMRS, Balas VE, Iliyasu AM (eds) Computational vision and bio-inspired computing. ICCVBIC 2019. Advances in intelligent systems and computing, vol 1108. Springer, Cham, pp 321–328. 10.1007/978-3-030-37218-7_37

[40] Rakshit S, Manna S, Biswas S, Kundu R, Gupta P, Maitra S et al (2017) Prediction of diabetes type-II using a two-class neural network. In: Mandal JK, Dutta P, Mukhopadhyay S (eds) Computational intelligence, communications, and business analytics. First international conference, CICBA 2017, March 2017. Communications in computer and information science, vol 776. Springer, Singapore, 65-71. 10.1007/978-981-10-6430-2_6

[41] Sapon MA, Ismail K, Zainudin S (2011) Prediction of diabetes by using artificial neural network. In: Abstracts of 2011 international conference on circuits, system and simulation IPCSIT vol. 7, IACSIT Press, Singapore, 28 May 2011

[42] Shanker MS (1996). Using neural networks to predict the onset of diabetes mellitus. J Chem Inf Comput Sci.

[43] Temurtas H, Yumusak N, Temurtas F (2009). A comparative study on diabetes disease diagnosis using neural networks. Expert Syst Appl.

[44] Kumar A, Gupta PK, Srivastava A (2020). A review of modern technologies for tackling COVID-19 pandemic. Diabetes Metab Syndr: Clin Res Rev.

[45] Yahyaoui A, Jamil A, Rasheed J, Yesiltepe M (2019) A decision support system for diabetes prediction using machine learning and deep learning techniques. In: Abstracts of the 1st international informatics and software engineering conference, IEEE, Ankara, 6-7 November 2019. 10.1109/UBMYK48245.2019.8965556

[46] Prabhu P, Selvabharathi S (2019) Deep belief neural network model for prediction of diabetes mellitus. In: Abstracts of the 3rd international conference on imaging, signal processing and communication, IEEE, Singapore, 27-29 July 2019. 10.1109/ICISPC.2019.8935838

[47] Idriss TE, Idri A, Abnane I, Bakkoury Z (2019) Predicting blood glucose using an LSTM neural network. In: Abstracts of 2019 federated conference on computer science and information systems, IEEE, Leipzig, 1-4 September 2019. 10.15439/2019F159

[48] Jankovic MV, Mosimann S, Bally L, Stettler C, Mougiakakou S, Belgrade IEEE (2016) 22-24 November 2016. 10.1109/NEUREL.2016.7800095

[49] Song W, Cai WY, Li J, Jiang FS, He SQ (2019) Predicting blood glucose levels with EMD and LSTM based CGM data. In: Abstracts of the 6th international conference on systems and informatics, IEEE, Shanghai, 2-4 November 2019. 10.1109/ICSAI48974.2019.9010318

[50] Zhang L, Zhu F, Xie L, Wang C, Wang J, Chen R (2020). Clinical characteristics of COVID-19-infected cancer patients: a retrospective case study in three hospitals within Wuhan, China. Ann Oncol.

[51] Marco ML, Heeney D, Binda S, Cifelli CJ, Cotter PD, Foligné B (2017). Health benefits of fermented foods: microbiota and beyond. Curr Opin Biotechnol.

[52] Wu JH, Li J, Wang J, Zhang L, Wang HD, Wang GL (2020). Risk prediction of type 2 diabetes in steel workers based on convolutional neural network. Neural Comput Appl.

[53] Wang LY, Mu Y, Zhao J, Wang XY, Che HL (2020). IGRNet: a deep learning model for non-invasive, real-time diagnosis of prediabetes through electrocardiograms. Sensors (Basel).

[54] Ayon SI, Islam M (2019). Diabetes prediction: a deep learning approach. Int J Inf Eng Electron Bus.

[55] Alhassan Z, McGough AS, Alshammari R, Daghstani T, Budgen D, Moubayed NA (2018) Type-2 diabetes mellitus diagnosis from time series clinical data using deep learning models. In: Kůrková V, Manolopoulos Y, Hammer B, Iliadis L, Maglogiannis I (eds) Artificial neural networks and machine learning - ICANN 2018. 27th international conference on artificial neural networks, October 2018. Lecture notes in computer science, vol 11141. Springer, Cham. 10.1007/978-3-030-01424-7_46

[56] Kumar NM, Manjula R, Satapathy SC, Bhateja V, Das S (2019). Design of multi-layer perceptron for the diagnosis of diabetes mellitus using keras in deep learning. Smart intelligent computing and applications. Smart innovation, systems and technologies.

[57] Mahajan AS (2020). Medical diagnosis of diabetes using deep learning techniques and big data analytics. J Emerg Technol Innov Res.

[58] Deshmukh T, Fadewar HS, Shukla A (2020) The detection of Prameha (diabetes) in Ayurvedic way with the help of fuzzy deep learning. In: Gunjan VK, Diaz VG, Cardona M, Solanki VK, Sunitha KVN (eds) ICICCT 2019 - System reliability, quality control, safety, maintenance and management. Springer, Singapore. 10.1007/978-981-13-8461-5_17

